# Ponicidin Induces Apoptosis via JAK2 and STAT3 Signaling Pathways in Gastric Carcinoma

**DOI:** 10.3390/ijms16011576

**Published:** 2015-01-12

**Authors:** Yuan-Fei Liu, Yun-Min Lu, Guo-Qiang Qu, Yuan Liu, Wei-Xiong Chen, Xiao-Hong Liao, Wu-Ming Kong

**Affiliations:** Department of Gastroenterology, Shanghai Jiao Tong University Affiliated Sixth People’s Hospital, Shanghai 200233, China; E-Mails: wangyang163jed@163.com (Y.-F.L); 203230@sjtu.edu.cn (Y.-M.L.); wyjedsw@163.com (G.-Q.Q.); wyjrdsw@163.com (Y.L.); wyjrdwz@163.com (W.-X.C.); wyjedwz@163.com (X.-H.L.)

**Keywords:** ponicidin, gastric carcinoma, MKN28 cells, apoptosis, JAK2, STAT3

## Abstract

Ponicidin has a variety of biological effects such as immunoregulatory and anti-inflammatory functions as well as anti-viral functions especially in the upper respiratory tract infection. This study was aimed to elucidate the antitumor effect of ponicidin in gastric carcinoma MKN28 cells and the possible molecular mechanism involved. Cell viability was measured by the Cell Count Kit-8 (CCK8). Cell apoptosis was assessed by flow cytometry as well as cell cycle and reactive oxygen species (ROS) analysis. Western blot analysis was used to detect the active form of caspase-3 as well as Bax and B-cell lymphoma-2 (Bcl-2) expressions after cells were treated with different concentrations of ponicidin. The results revealed that ponicidin could inhibit the growth of MKN28 cells significantly in both a time- and dose-dependent manner. The cell cycle was blocked and ROS generation was increased after the cells were treated with ponicidin. Bcl-2 expression was down-regulated remarkably while Bax expression and the active form of caspase-3 were increased after apoptosis occurred. We therefore conclude that ponicidin exhibited significant growth inhibition of gastric carcinoma cell line MKN28 and induced apoptosis of MKN28 cells via the signaling pathway regulated by Janus kinase 2 (JAK2) and signal transducers and activators of transcription 3 (STAT3). Ponicidin may serve as a potential therapeutic agent for gastric carcinoma.

## 1. Introduction

Gastric carcinoma (GC) is the fourth most common cancer and the second highest cause of cancer-related mortality worldwide [1]. The prognosis of patients with GC continues to be dismal, despite improving surgical and adjuvant treatment approaches, with a five-year overall survival less than 25% [2]. It is of great clinical importance to identify genes that control the severity of GC and demonstrate predictive value for prognosis [3,4].

During physiological and pathological angiogenesis, vascular endothelial growth factor (VEGF) plays a unique role for vascular endothelial cells [5]. Vascular endothelial growth factor receptor 2 (VEGFR2) is the primary receptor of VEGF and the major mediator of VEGF-induced angiogenesis pathways including signal transducers and activators of transcription (STATs) [6,7]. Recently many studies showed the important roles of VEGFR2 in potential drug discovery and molecular mechanism research [8,9,10]. In addition, increasing evidence shows that withinVEGFR2-mediated signaling, the Janus kinase 2 (JAK)/STAT signaling pathway, especially STAT3, is the critical target and biomarker during angiogenesis and tumor growth. JAK/STAT-mediated apoptosis can be found in gastric carcinoma [11], hepatocellular carcinoma [12], and colorectal cancer [13], especially through the phosphorylation of STAT3 to promote survival and inhibit apoptosis.

Ponicidin, a diterpenoid compound, is extracted and purified from the traditional Chinese herbs *Rabdosia rubescens* or *Isodon japonicas* [14,15]. Ponicidin has been reported to have a variety of biological effects such as immunoregulatory and anti-inflammatory functions as well as anti-viral functions especially in upper respiratory tract infection [16]. Recent laboratory data suggest that ponicidin is a very effective anti-tumor agent, with profound effects on a number of cancer cells such as human leukemia cell line K562, human breast cancer cell line Bcap37, human gastric cancer cell line BGC823, human urinary bladder cancer cell line BIU87, and HeLa cell lines [14,15,16,17]. New data have demonstrated that ponicidin can inhibit the growth and metastasis of prostate cancer due to its significant antiangiogenic activity [18].

Although ponicidin has been proved to be very effective in a variety of malignancies, many of its anti-tumor mechanisms remain to be demonstrated. To date, no detailed data are available about the role and mechanisms of ponicidin in gastric carcinoma cells. In order to understand the roles of ponicidin in gastric carcinoma cells and possible clinical application of ponicidin in gastric carcinoma therapy, we have investigated the effects of different concentrations of ponicidin on apoptosis of gastric carcinoma cell MKN28 and elucidated the possible molecular mechanisms involved.

## 2. Results and Discussion

### 2.1. Ponicidin Inhibits Proliferation of MKN28 Cells

**Figure 1:**
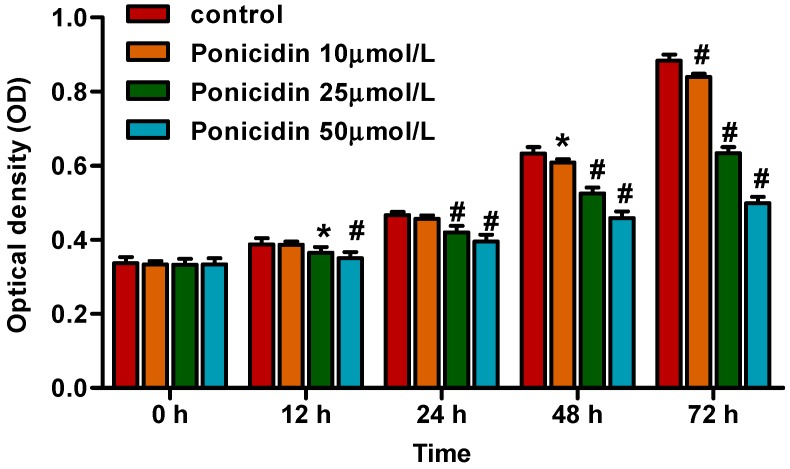
Ponicidin inhibited the viability of MKN28 cells. Cell viability was measured by the Cell Count Kit-8 (CCK8). Ponicidin (10, 25 and 50 μmol/L) significantly inhibited MKN28 cells viability in a time- and dose-dependent manner when compared with the control group. Date are presented as mean ± SD (standard deviation) (*n* = 3). * *p* < 0.05, # *p* < 0.01.

To investigate the growth inhibition effects of ponicidin on MKN28 cells, cell viability was evaluated by CCK8 after treatment with various concentrations of ponicidin for 0, 12, 24, 48 and 72 h. As shown in Figure 1, ponicidin had significant growth inhibition effects on the MKN28 cells in a dose-and time-dependent manner. Cell viability was decreased remarkably after the cells were treated with ponicidin at 10 μmol/L for 48 h.

### 2.2. Ponicidin Induces Apoptosis of MKN28 Cells

**Figure 2:**
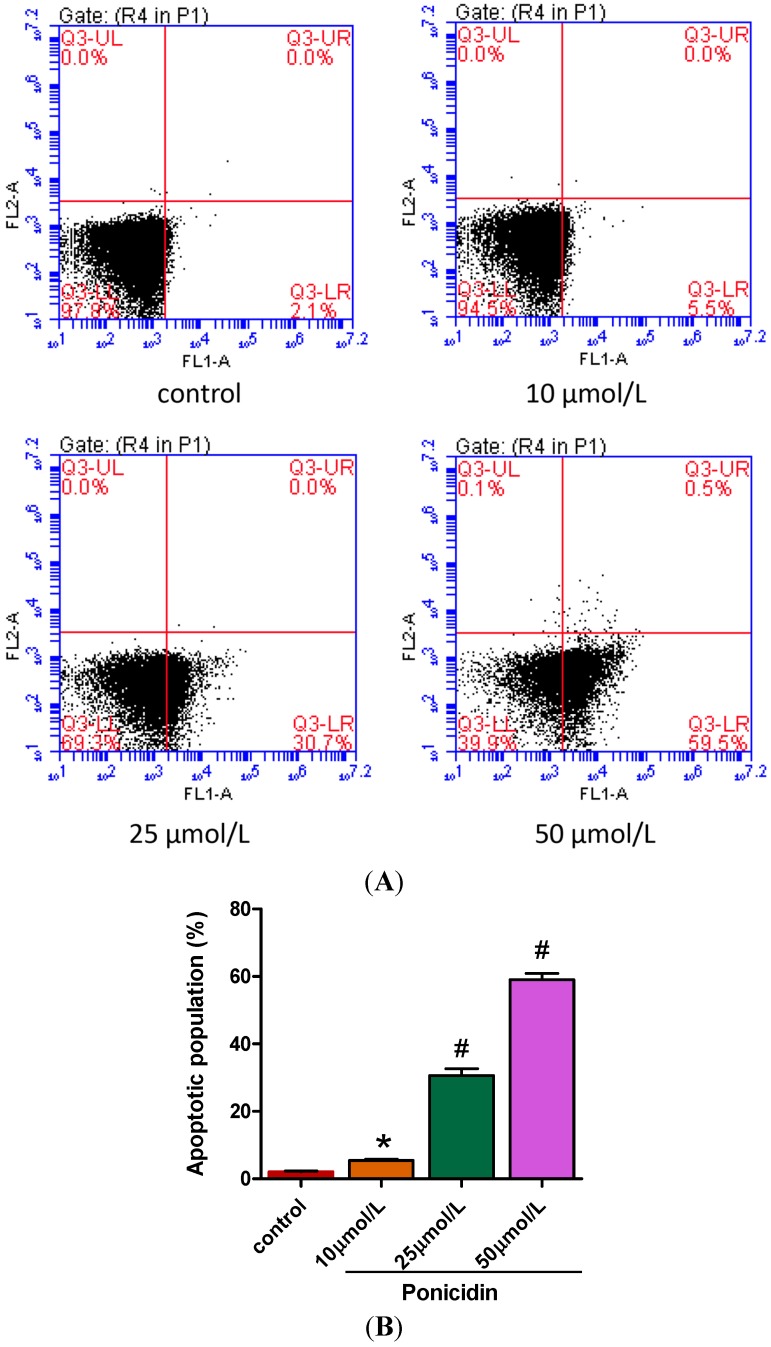
Effects of ponicidin on apoptosis of MKN28 cells. (**A**) Annexin-V/PI double stain assay and flow cytometry analysis were carried out to substantiate cell apoptosis; and (**B**) Treatment of ponicidin at doses of 10, 25 and 50 μmol/L for 48 h dose-dependently increased the apoptotic populationof MKN28 cells. The lower left quadrant shows vital cells. The lower right quadrant indicates early apoptotic cells (Annexin-V positive but PI negative). The upper right quadrant represents late apoptotic cells or necrotic cells (double positive). * *p* < 0.05, # *p* < 0.01.

An annexin-V fluorescein isothiocyanate (FITC)/propidium iodide (PI) double stain assay and flow cytometry analysis were carried out to substantiate cell apoptosis induced by ponicidin treatment under various concentrations. The number of apoptotic cells was counted as late apoptotic cells shown in theupper right quadrant and early apoptotic cells as shown in lower right quadrant of the histograms. As shown in Figure 2, treatment of ponicidin at the dose of 10, 25 and 50 μmol/L for 48 h significantly increased the number of early apoptotic cells, respectively, from 2.13% ± 0.15% to 59.03% ± 1.84% (*n* = 3) in a dose-dependent manner compared with control cells with that of 2.13% ± 0.15%. The significant induction of apoptosis indicated the anticancer effect of ponicidin against MKN28 cells.

### 2.3. Ponicidin Arrests the Cell Cycle of MKN28 Cells

**Figure 3:**
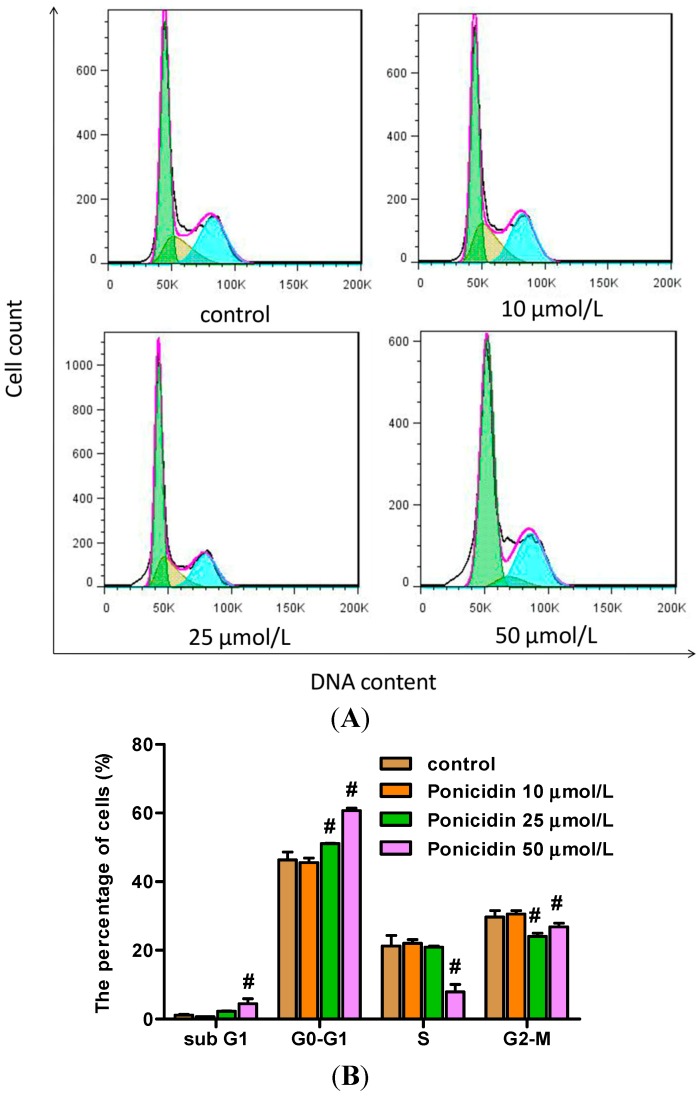
Effects of ponicidin on the cell cycle of MKN28 cells. (**A**) PI stain assay and flow cytometry were carried out to analyze the cell cycle after MKN28 cells were treated with ponicidin at the dose of 10, 25 and 50 μmol/L for 48 h; and (**B**) The percentages of sub G1 and G0–G1 phase cells in MKN28 cells were increased while the percentages of S and G2–M phase cells were decreased in a dose-dependent manner especially treated with 50 μmol/L ponicidin. # *p* < 0.01.

MKN28 cells were treated with different concentrations of ponicidin for 48 h, and then stained with PI and analyzed by flow cytometry. As shown in Figure 3, the percentage of G0–G1 phase cells in the cell line was increased in a dose-dependent manner, especially treated with 25 and 50 μmol/L ponicidin, respectively, from 51.09% ± 0.15% to 60.68% ± 0.78% (*n* = 3) compared with control cells at 46.40% ± 2.26%. The percentage of MKN28 cells in sub G1 phase was also increased in dose-dependent manner, particularly when treated with 50 μmol/L ponicidin for 48 h. However the percentage of G2–M phase cells in the cell line decreased after treatment with 25 and 50 μmol/L ponicidin, respectively, from 24.03% ± 0.91% to 26.85% ± 1.02% (*n* = 3) compared with control cells at 29.71% ± 1.88%. The percentage of S phase cells in the cell line was also decreased after the treatment with 50 μmol/L ponicidin.

### 2.4. Ponicidin Induces Reactive Oxygen Species (ROS) Generation in MKN28 Cells

**Figure 4:**
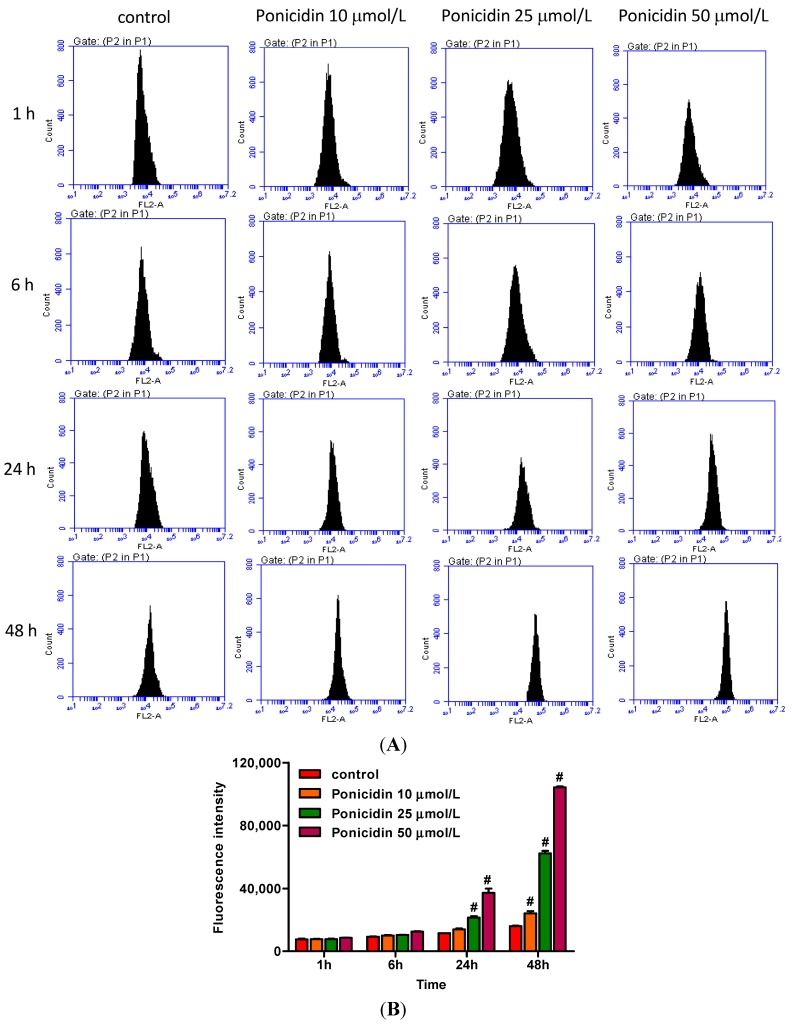
Effects of ponicidin on ROS generation in MKN28 cells. (**A**) After treatment with ponicidin at doses of 10, 25 and 50 μmol/L for 1, 6, 24 and 48 h, cells were resuspended with 50 μmol/L DHE and analyzed by flow cytometry; and (**B**) Ponicidin treatment time- and dose-dependently increased ROS generation of MKN28 cells. # *p* < 0.01.

After treatment as described in Section 3.4., cells were resuspended with dihydroethidium (DHE) and analyzed by flow cytometry. As shown in Figure 4, treatment of ponicidin at doses of 10, 25 and 50 μmol/L for different times significantly increased the fluorescence intensity of the MKN28 cells, respectively, from 7997.13% ± 335.87% to 2,102,040% ± 62,200.04% (*n* = 3) in a time- and dose-dependent manner compared with control cells with 2826.07% ± 195.51%, and significantly increased in 48 h.

### 2.5. Ponicidin Effects on Proteins Expression in MKN28 Cells

**Figure 5:**
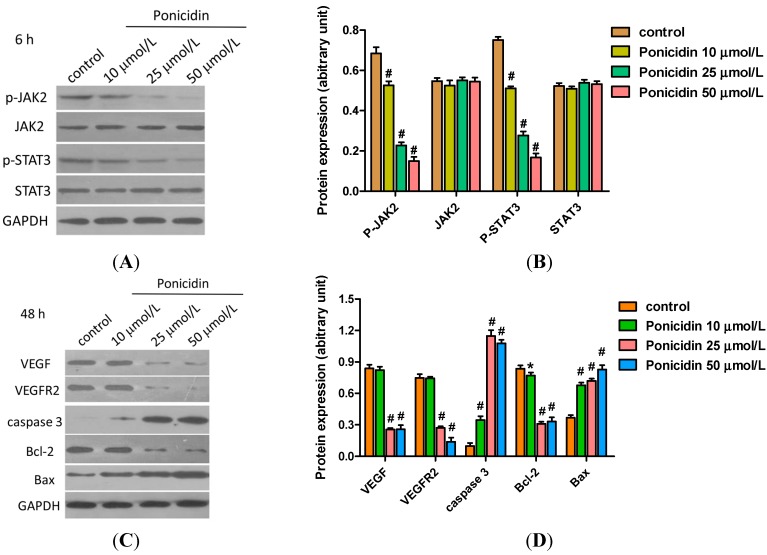
Effects of ponicidin on the expression of key proteins involved in cell apoptosis. (**A,C**) The expression of apoptosis-related proteins was detected by Western blot; (**B**) Treatment with ponicidin for 6 h: JAK2 and STAT3 phosphorylation decreased dose-dependently and no effects of ponicidin (50 μg/mL) on JAK2 and STAT3 were observed; and (**D**) Treatment with ponicidin (25, 50 μg/mL) for 48 h increased the expression of Bax and the active form of caspase-3, while it decreased the expression of VEGF, VEGFR2 and Bcl-2, dose-dependently. * *p* < 0.05, # *p* < 0.01.

To clarify the mechanism of MKN28 cells apoptosis induced by ponicidin, the expression of apoptosis-related proteins and the phosphorylation of kinases were detected by western blot. As shown in Figure 5A, treatment with ponicidin (10, 25 and 50 μmol/L) for 6 h decreased JAK2 phosphorylation and STAT3 phosphorylation in a dose-dependent manner, while MKN28 cells treated with ponicidin had no effect on the protein levels of JAK2 and STAT3. Treatment with ponicidin (25, 50 μmol/L) for 48 h also evidently decreased the protein levels of VEGF, VEGFR2 and Bcl-2 in a dose-dependent manner compared to the control group, but increased levels of Bax protein and the active form of caspase-3 (Figure 5B).

### 2.6. Ponicidin Induces Activity of Caspase-3 in MKN28 Cells

**Figure 6:**
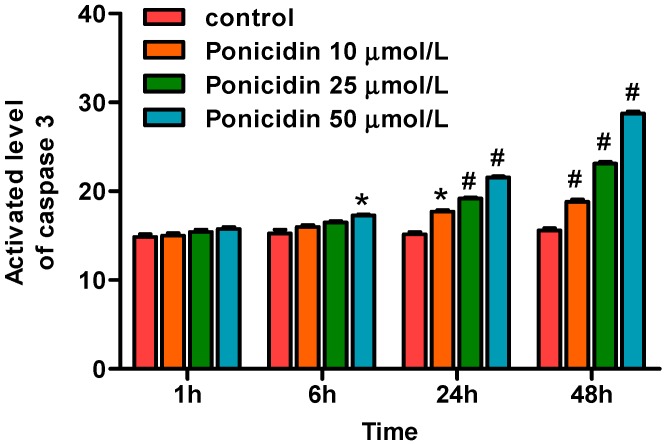
Effects of ponicidin on the activity of caspase-3 in MKN28 cells. After treatment, the activity of caspase-3 in MKN28 cells was analyzed by the caspase-3 assay kit. Ponicidin treatment time- and dose-dependently increased the activity of caspase-3 of MKN28 cells. * *p* < 0.05, # *p* < 0.01.

To clarify the effect of ponicidin on caspase-3, we also investigated the activity of caspase-3 after ponicidin treatment. As shown in Figure 6, treatment of MKN28 cells with ponicidin (10, 25 and 50 μmol/L) for different times induced caspase-3 activation respectively, from 18.80% ± 0.47% to 28.76% ± 0.38% (*n* = 3) in a time- and dose-dependent manner compared with control cells with that of 15.57% ± 0.47% and significantly increased in 48 h.

Gastric carcinoma is estimated to be the world’s second most common cancer. Because the incidence of gastric carcinoma can change dramatically from region to region and from one generation to the next, it has been hypothesized that its incidence is determined largely by environmental rather than genetic factors [19]. *Helicobacter pylori* has been linked to chronic atrophic gastritis, an established precursor of the intestinal type of gastric carcinoma [20,21]. High rates of infection have also been found in patients with cancer or precancerous conditions [22], and a case-control study found that patients with gastric carcinoma had a relatively high risk of *H. pylori* infection compared to healthy control subjects [23].

Despite recent advances in our understanding of the molecular biology of gastric carcinoma cells and the induction of some new chemotherapeutic agents for the treatment of this malignant disease, there are few efficient therapeutic measures or regimes. Therefore, it is a permanent challenge to find new drugs and effective therapies for the clinical treatment of gastric carcinoma.

In this study, we found that ponicidin, a diterpenoid compound extracted from traditional Chinese herbs, could inhibit MKN28 cell proliferation in a dose-dependent manner. The result of flow cytometry analysis by annexin V/PI staining showed that ponicidin treatment from 10 to 50 μmol/L time- and dose-dependently induced apoptosis and the percentages of sub G1 and G0–G1 phase cells of MKN28 cells were increased, while S and G2–M phase cells were decreased in a dose-dependent manner after the cells were treated with ponicidin for 48 h. These results indicate that ponicidin could remarkably inhibit the proliferation of MKN28 cells, block the cell cycle, and facilitate the apoptosis of MKN28 cells. ROS generation has been shown to be a common cellular mechanism for multiple cell death pathways [24], including gene activation, cell cycle arrest and apoptosis. In addition, ROS are directly microbiocidal and are important for amplifying pro-inflammatory pathways, such as NF-κB and JNK [25]. In this study, ponicidin promoted both apoptosis and ROS generation in MKN28 cells. The results showed that ponicidin induced the disruption of the mitochondrial transmembrane potential followed by the production of ROS that later could lead to the onset of apoptosis. Furthermore, caspase activation plays a central role in the execution of apoptosis. The key components of the biochemical pathways of caspase activation have been recently elucidated, and there are two most well-studied pathways of caspase activation: the cell surface death receptor pathway and the mitochondria-initiated pathway [26]. In this study, we report for the first time that ponicidin induced caspase-3 activation in a time- and dose-dependent manner; these data suggest that ponicidin may be used as an effective apoptosis inducer on gastric carcinoma cells *in vitro*.

Recently medicinal plants and prescriptions were reported to show antitumor effects via antiangiogenic activity. For instance, decursin isolated from *Angelica gigas* Nakai exerted the anticancer activities in breast and prostate cancer cells [27,28]. *Panax ginseng* C.A MEYER is a well-known anti-cancer and chemopreventive agent [29] and Kamikaekyuktang also exhibited antitumor activity via apoptosis and antiangiogenesis [30]. Ponicidin was recently reported to be very effective in inducing growth inhibition and apoptosis in a variety of malignant cell lines [31,32]. In our study, we found that the protein levels of VEGF, VEGFR2, JAK2 phosphorylation and STAT3 phosphorylation were decreased after cells were treated with 50 μmol/L ponicidin. This suggests that ponicidin functioned as an antitumor agent via suppression of the VEGFR2-mediated JAK2-STAT3 signaling pathway. JAK2, a member of the Janus (JAK) family of non-receptor protein tyrosine kinases, regulates signaling via multiple cytokine receptors [33,34]. STAT3, which is associated with oncogenesis, cell proliferation, angiogenesis, immune evasion, and apoptotic resistance, is constitutively activated in human hepatocellular carcinoma tissues, but not in normal human liver tissues [35,36,37].

Apoptosis play an important role in the development and maintenance of homeostasis with all multicellular organisms, and impaired apoptosis is now recognized to be a key step in tumorigenesis [38]. It was considered an important method of assessment for the clinical effectiveness of many anti-tumor drugs and a significant index for the selection of new anti-tumor drugs [39,40]. Bcl-2 represents the founding member of the new and growing class of cell death inhibiting oncoproteins [41]. The first pro-apoptotic homolog of the Bcl-2 family, Bax, was identified by coimmunoprecipitation with Bcl-2 protein. When Bax was overexpressed in cells, apoptotic death in response to death signals was accelerated, earning its designation as a death agonist. When Bcl-2 was overexpressed, it heterodimerized with Bax and death was repressed, thus the ratio of Bcl-2 to Bax is important in determining susceptibility to apoptosis [42]. In this study, our results revealed that Bcl-2 expression was down-regulated remarkably while Bax expression and the active form of caspase-3 were up-regulated after apoptosis occurred, and the ratio of Bcl-2 to Bax was down-regulated: apoptosis therefore was induced in ponicidin treated gastric carcinoma cells. The data suggest that ponicidin may serve as a potential therapeutic agent for gastric carcinoma. The *in vivo* antitumor effects of ponicidin as well as its potential clinical effectiveness need further and thorough investigation.

## 3. Experimental Section

### 3.1. Reagents

Ponicidin was kindly procured from Nanjing Zelang Medical Technological Co., Ltd. (Nanjing, China). Antibodies for JAK2, P-JAK2, STAT3, P-STAT3 were purchased from Cell Signaling Technology, Inc. (Beverley, MA, USA) and GAPDH from Fermentas (Vilnius, LT, USA). Antibodies for VEGF, VERFR2, caspase-3, Bcl-2 and Bax were purchased from Abcam (Cambridge, MA, USA).

### 3.2. Cell Culture

The human MKN28 cell line was obtained from Shanghai Institute of Cell Biology (Shanghai, China). Cells were cultured in RPMI-1640 medium with 10% FBS (Gibco BRL, Rockville, MD, USA), 100 U/mL penicillin G and 100 μg/mL streptomycin in an incubator (37 °C, 100% humidity and 5% CO_2_, Thermo, Fisher Scientific Inc., Waltham, MA, USA).

### 3.3. Cell Viability Assay

The Cell Count Kit-8 (CCK8, Dojindo, Rockville, MD, USA, http://www.dojindo.com) was used to assess the effects of ponicidin on cell viability [43]. In brief, MKN28 cells in logarithmic growth-phase were collected, and 5 × 10^3^cells/well were dispensed into 96-well culture plates with 100 μL culture medium. After 24 h culture different concentrations of ponicidin (10, 25 and 50 μmol/L) were put in different wells. Each of the concentrations above was regarded as one treated group while there was no ponicidin in the control group. Each treated or control group contained three parallel wells. Culture plates were then incubated for 0, 12, 24, 48 and 72 h. Subsequently the cell viability was evaluated by CCK8 following the manufacturer’s instructions. The absorbance at wavelength 450 nm was measured for the supernatant of each well using the plate reader Multiskan EX (Thermo Fisher Scientific Inc., Waltham, MA, USA; http://www.thermofisher.com).

### 3.4. Flow Cytometry (FCM) Detection

Apoptosis was determined by flow cytometry analysis. MKN28 cells were collected after treatment with ponicidin (10, 25 and 50 μmol/L) for 48 h. Annexin-V fluorescein isothiocyanate (FITC)/propidium iodide (PI) double stain assays (Biovision Inc, Mountain View, CA, USA) were performed following the manufacturer’s protocol. Both floating and trypsinized adherent cells were collected, resuspended in 500 μL of binding buffer containing 2.5 μL of annexin-V FITC and 5 μL of propidium iodide (PI), and then incubated for 10 min in the dark at room temperature before flow cytometry analysis.

Cell cycles were examined using PI and flow cytometry. MKN28 cells were seeded in 12-well plates at the density of 3 × 10^3^ cells/well and then treated with 10, 25 and 50 μmol/L of ponicidin for 48 h. After treatments, the percentages of cells in the different phases of cell cycle were evaluated by determining the DNA content after PI staining. Briefly, cells were washed with PBS, trypsinized and centrifuged at 400× *g* at 4 °C for 5 min. Pellets were fixed overnight in 70% cold ethanol. After fixation, cells were washed twice with PBS and incubated in PBS containing RNase (1 mg/mL) for 10 min at room temperature. Finally, samples were stained with propidium iodide (1 mg/mL) for 30 min at 4 °C. Data acquisition was done by flow cytometry (Epics XLM.CL, Beckman Coulter, Fullerton, CA, USA) using Cell Quest software (BD, Franklin Lakes, NJ, USA).

Intracellular generation of ROS was detected using the fluorescent probe dihydroethidium (DHE). DHE is a poorly fluorescent 2-electron reduction product of ethidium that on oxidation produces DNA sensitive fluorochromes that generate a red nuclear fluorescence when excited at 510 nm. The results obtained with this probe were validated as a measure of the ability of MKN28 cells to generate ROS. After treatment with ponicidin at doses of 10, 25 and 50 μmol/L for 1, 6, 24 and 48 h, cells were resuspended with 50 μmol/L DHE, and fluorescence intensity was measured using flow cytometry.

### 3.5. Western Blot

Following treatment with ponicidin at the desired concentrations and time, MKN28 cells were harvested. The expression of apoptosis-related proteins was detected by Western blot according to the manufacturer’s instructions and previous reports [44]. Glyceraldehyde 3-phosphate dehydrogenase (GAPDH) antibody was used as an internal control for whole cell lysates. The experiment was repeated three times independently.

### 3.6. Caspase-3 Activation Assay

The activity of caspase-3 was analyzed by the caspase-3 colorimetric assay Kit (KeyGEN, Nanjing, China) following the manufacturer’s protocol. Briefly, MKN28 cells were collected, resuspended in 50 μL of chilled cell lysis buffer and incubated on ice for 10 min. After centrifugation for 1 min (at 400× *g*), the supernatant was transferred to a fresh tube and then 100 μg protein was diluted in 50 μL cell lysis buffer for each assay. Samples were read at 405 nm in a plate reader Multiskan EX (Labsystems, Helsinki, Finland).

### 3.7. Statistical Analysis

Values were expressed as means ± SD (standard deviation). One-way analysis of variance (ANOVA) followed by Dunnett’s test was used for statistical analysis. Probability (*p*) values less than 0.05 were considered significant.

## 4. Conclusions

Ponicidin has significant anti-proliferation effects by inducing apoptosis on gastric carcinoma cells *in vitro*, and induced apoptosis of MKN28 cells via the pathway regulated by JAK2 and STAT3 signaling pathways. To our knowledge, this is the first report concerning the roles of ponicidin on gastric carcinoma cells *in vitro*; our results suggest that ponicidin may serve as a potential therapeutic agent for gastric carcinoma.
